# Sauchinone inhibits the proliferation, migration and invasion of breast cancer cells by suppressing Akt-CREB-MMP13 signaling pathway

**DOI:** 10.1042/BSR20211067

**Published:** 2021-10-28

**Authors:** Na Hui Kim, Nam Ji Sung, Seokwon Shin, Deok-Seon Ryu, Hyung-Sun Youn, Sin-Aye Park

**Affiliations:** 1 Department of ICT Environmental Health System, Graduate School, Soonchunhyang University, Asan-si 31538, Republic of Korea; 2 Department of Biomedical Laboratory Science, College of Medical Sciences, Soonchunhyang University, Asan-si 31538, Republic of Korea

**Keywords:** Breast cancer cells, CREB, matrix metalloproteinase-13, Sauchinone

## Abstract

Sauchinone, a lignan isolated from *Saururus chinenesis*, is known to exhibit anti-inflammatory and anti-oxidant effects. Recently, sauchinone has been reported to inhibit the growth of various cancer cells, but its effects on breast cancer cells remain poorly understood. In the present study, we investigated the effects of sauchinone on the growth of breast cancer cells along with the underlying molecular mechanisms. Our results show that sauchinone treatment markedly inhibited the proliferation, migration, and invasion of breast cancer cells. Sauchinone reduced the phosphorylation of Akt, ERK, and CREB increased by transforming growth factor-β (TGF-β). In particular, sauchinone treatment suppressed the expression of matrix metalloproteinase (MMP)-13 (MMP13) by regulating the Akt-CREB signaling pathway. Sauchinone was less effective in inhibiting cell migration in *Mmp13*-knockdown cells than in control cells, suggesting that MMP13 may be a novel target for sauchinone. Our study suggests that sauchinone inhibits the growth of breast cancer cells by attenuating the Akt-CREB-MMP13 pathway. In addition, the targeted inhibition of MMP13 by sauchinone represents a promising approach for the treatment of breast cancer.

## Introduction

Breast cancer is one of the most common cancers among women worldwide [1]. Although breast cancer treatment has improved substantially, many patients continue to manifest serious side effects, treatment resistance, and risk of recurrence and metastasis [2]. Effective patient-specific treatment of breast cancer requires the discovery of a variety of therapeutic targets in addition to typical cellular targets such as estrogen receptors (ERs), progesterone receptors, and human epidermal growth factor receptor 2. In addition, effective therapeutic agents with few side effects and clear molecular targets are needed. Toward this end, it is important to evaluate the anti-cancer efficacy of natural ingredients and elucidate the underlying molecular mechanisms.

Sauchinone, a lignan isolated from *Saururus chinensis* (*Saururaceae*), is known to exhibit various pharmacological properties such as anti-inflammatory and anti-oxidant activities. Among the methanol extracts obtained from the roots of *S. chinensis*, sauchinone and sauchinone B decreased the plasma levels of tumor necrosis factor-α and alanine aminotransferase induced by lipopolysaccharide (LPS)/d-galactosamine, showing the strongest protective effects against lethality in mice [3]. Sauchinone suppressed the gene expression of inflammatory cytokines such as interleukin (IL)-5 and IL-13, consequently suppressing allergic airway inflammation in a murine model of allergic asthma [4]. In addition, sauchinone has been reported to inhibit the inflammatory response in acute lung injury [5], osteoarthritis [6,7], and colitis [8,9]. The anti-inflammatory effect of sauchinone is attributed to its anti-oxidant activity. Sauchinone suppressed the production of intracellular radicals and increased the anti-oxidant enzymes catalase and superoxide dismutase [10]. Its protective effect against oxidative stress was also induced by up-regulating Nrf2-dependent heme oxygenase-1 (HO-1) expression [11]. Sauchinone inhibited pro-inflammatory mediators by increasing the expression and activity of HO-1 in monocyte/macrophage-like cells such as RAW264.7 cells [12].

Increased evidence in recent years suggests that sauchinone exhibits anti-tumor effects. Sauchinone induced apoptosis in prostate and breast cancer cells via activation of caspase-3 [13]. Sauchinone suppressed the epithelial–mesenchymal transition (EMT) in pancreatic ductal adenocarcinoma cells [14] and gastric cancer cells [15]. Sauchinone has been reported to inhibit migration and invasion of hepatocellular carcinoma cells by targeting AMPK [16] or STAT3 [17] signaling pathway. In addition, the anti-cancer effect of sauchinone in lung adenocarcinoma cells was mediated via down-regulation of EIF4EBP1 [18]. Although many studies reported the anti-inflammatory, anti-oxidant and anti-cancer efficacy of sauchinone, its effects on breast cancer cells and their molecular mechanisms are still unknown.

Matrix metalloproteinases (MMPs) are zinc-dependent endopeptidases that induce EMT, cell migration, and metastasis [19]. The MMPs are highly expressed in breast cancer cells during breast cancer progression [20–22]. In microarray results, the mRNA expression of MMP1, -9, -11, -12, and -13 was increased in higher grades of breast cancer compared with normal breast tissues [23]. MMP9 synthesized in breast cancer cells is important for invasion and lung metastasis in a mouse orthotopic model of basal-like breast cancer [24]. Among MMPs, MMP13 is well known for its effects on breast cancer progression. MMP13 is one of the secreted proteins overexpressed in breast cancer tissues compared with normal adjacent tissues [25]. Overexpression of MMP13 at the tumor-bone interface triggered mammary tumor-induced osteolysis [26]. MMP13 was involved in breast cancer progression induced by ETV4 transcription factor [27], Golgi membrane protein 1 [28], and gremlin-1 (GREM1) [29]. These findings suggest that MMP13 is a major molecular target in breast cancer growth.

In the present study, we investigated whether sauchinone exerts anti-tumor effects in breast cancer cells. Our results show that sauchinone attenuated the proliferation, migration, and invasion of breast cancer cells by suppressing Akt-CREB-MMP13 signaling pathway.

## Materials and methods

### Cell culture and reagents

**Figure 1:**
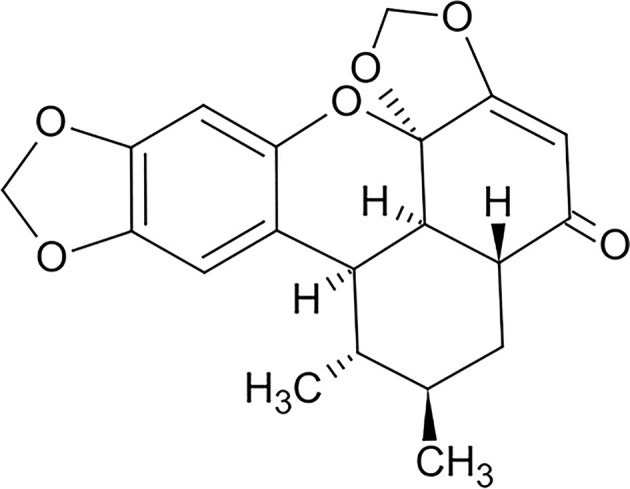
Chemical structure of sauchinone

MDA-MB-231 and MTV/TM-011 cells were originally obtained from American Type Culture Collection and Korean Cell Line Bank. MDA-MB-231 cells were cultured in DMEM (Corning Inc., NY, U.S.A.) containing 10% fetal bovine serum (FBS, Thermo Fisher Scientific, Waltham, MA, U.S.A.) and 1% penicillin/streptomycin (Corning Inc.). MTV/TM-011 cells were cultured in RPMI (Corning Inc.) containing 10% FBS and 1% penicillin/streptomycin. Cells were maintained at 37°C in a humidified atmosphere with 5% CO_2_/95% air. Sauchinone (Cat#. SML0783) (Figure 1) and 666-15 (Cat#. 538341) were purchased from Sigma–Aldrich (St. Louis, MO, U.S.A.). Recombinant human/mouse transforming growth factor-β1 (TGF-β1) were obtained from SinoBiological (Beijing, China, Cat#. 10804-HNAC/50698-M08H). Anti-phospho-STAT3 (Cat#. 9145), anti-STAT3 (Cat#. 9139), anti-phospho-CREB (Cat#. 9198), anti-CREB (Cat#. 9197), anti-phospho-ERK (Cat#. 4370), anti-ERK (Cat#. 4695), anti-phospho-Akt (Cat#. 4060), anti-Akt (Cat#. 4691), anti-N-cadherin (Cat#. 13116), anti-Twist (Cat#. 46702), anti-Slug (Cat#. 9585), anti-MMP2 (Cat#. 40994), anti-MMP3 (Cat#. 14351), and anti-MMP9 (Cat#.13667) were obtained from Cell Signaling Technology (Danvers, MA, U.S.A.). Anti-MMP13 (sc-515284) and anti-β-actin (sc-47778) antibody were purchased from Santa Cruz Biotechnology (Dallas, TX, U.S.A.), U0126 (Cat#. S1102) and LY294002 (Cat#. S1105) were obtained from Selleckchem (Houston, TX, U.S.A.).

### Gene silencing

Endogenous MMP13 was knocked down using specific small interfering RNAs (siRNAs) (Bioneer, Daejeon, Korea, Cat#. 17386). Briefly, cells were transiently transfected with siRNAs by reverse transfection using Lipofectamine RNAiMAX (Invitrogen, Carlsbad, CA, U.S.A.).

### Western blot analysis

Standard sodium dodecyl sulfate/polyacrylamide gel electrophoresis (SDS/PAGE) and Western blotting were used to analyze the expression of various proteins. Cells were lysed in the lysis buffer (Cell Signaling Technology, Cat#. 9803) containing protease inhibitors and phosphatase inhibitors (Roche, Basel, Switzerland). The quantitative protein concentration was determined by BCA Protein Assay Kit (Thermo Fisher Scientific, Cat#. 23225) and equal amounts of protein were loaded on 8–12% SDS/PAGE. Proteins were transferred to polyvinylidene difluoride membrane (Merck Millipore, Burlington, MA, U.S.A.) and subjected to immunoblotting using various antibodies overnight at 4°C, followed by further incubation with the secondary antibody (AbFrontier, Seoul, Korea, Cat#. LF-SA8001 and LF-SA8002) at room temperature for 1 h. Visualization of protein bands was detected with Westsave Gold detection reagents (AbFrontier, Cat#. LF-QC0103).

### Cell proliferation assay

Cells were seeded in 96-well plates (1 × 10^4^/well) and incubated with DMSO or sauchinone for 2 days. The cells were then treated with 20 μl of CellTiter 96® Cell Proliferation Assay (Promega, Madison, WI, U.S.A., Cat#. G3582) for 2 h at 37°C. The absorbance of each well was detected at 490 nm with a Multiskan, GO microplate reader (Thermo Fisher Scientific). All procedures were performed according to the manufacturer’s instructions. For the colony formation assay, 1 × 10^3^ cells were plated in the 24-well plates, incubated with DMSO or sauchinone, and allowed to grow for 10–14 days. After the medium was removed, cells were fixed with 10% formalin for 15 min, and stained with Crystal Violet to visualize the colonies.

### Wound healing assay

Cells were seeded into 12-well culture dishes and wounded by manual scratching of the surface with a 1-ml pipette tip. The scratched surface was washed with PBS to remove cell debris. Dishes containing these cells were then treated with the medium containing each compound and incubated at 37°C for 48 h. Also, the cells were seeded at the density of 3 × 10^5^/ml into Ibidi Culture Inserts (Ibidi, Gewerbehof, Germany). After incubation for 24 h, Ibidi Culture Inserts were gently removed. Cells were incubated with each compound at 37°C for an additional 48 h. Images of wound sites were captured at 0 h (control) and 48 h using an inverted microscope (40× total magnification). Each wound area was determined using ImageJ software.

### Invasion assay

MTV/TM-011 cells (1 × 10^5^ cells) were suspended in serum-free medium containing each compound and seeded into the upper transwell inserts (Corning Inc., Cat#. 354480). The lower chambers were filled with medium containing 20% FBS. After incubation for 30 h, the bottom of transwell inserts was fixed with cold methanol and stained with 0.5% Crystal Violet. The non-invaded cells were wiped off and the invaded cells were counted in four randomly selected fields using an inverted microscope (100× total magnification).

### Statistical analysis

Data were expressed as the mean ± SD of results obtained from at least three independent experiments. Significant differences were determined by a Student’s *t* test or one-way ANOVA. A *P*-value of less than 0.05 was considered to be statistically significant. *, *P* < 0.05; **, *P* < 0.01; and ***, *P* < 0.001.

## Results

### Sauchinone suppresses breast cancer cell growth

**Figure 2:**
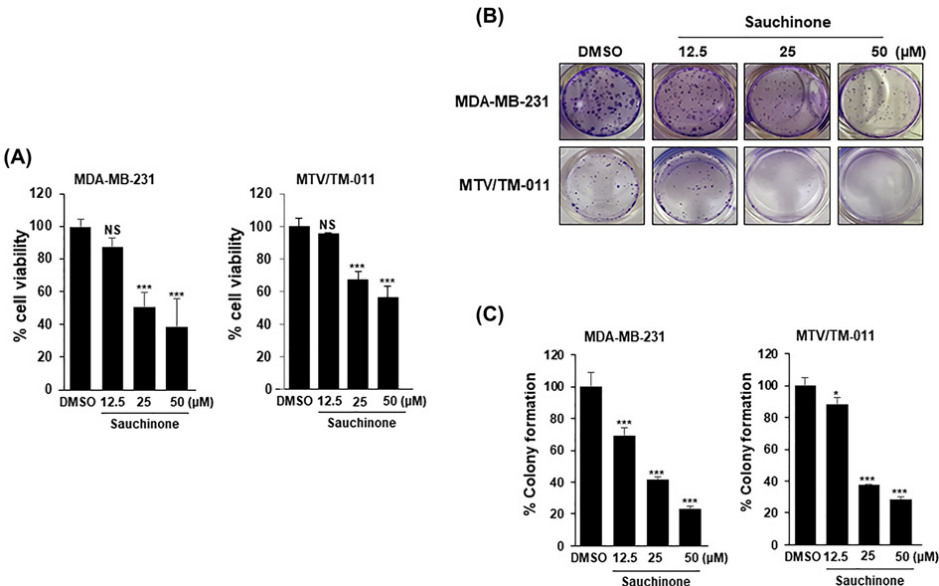
Effect of sauchinone on breast cancer cell growth (**A**) Cell growth was determined after treatment with or without 12.5, 25, and 50 µM sauchinone for 48 h. The cell viability was measured using MTS assay. (**B**,**C**) MDA-MB-231 and MTV/TM-011 cells were seeded in 24-well plates (1 × 10^3^/well). After incubation for 24 h, sauchinone was treated at various concentrations (12.5, 25, and 50 µM) for 10–14 days. Data are presented using triplicate wells per group and statistical significance was determined by one-way ANOVA. *, *P* < 0.05; ***, *P* < 0.001; NS, not significant.

To evaluate the effect of sauchinone on cell viability, breast cancer cell lines (MDA-MB-231 and MTV/TM-011) were treated with various concentrations of sauchinone (12.5, 25, and 50 µM) and then analyzed via MTS assay. Sauchinone significantly inhibited the viability of breast cancer cells in a dose-dependent manner (Figure 2A). Next, a colony formation assay was performed to assess the effect of sauchinone on the proliferation of breast cancer cells. Specifically, sauchinone treatment at 25 and 50 µM concentrations markedly inhibited colony formation in both MDA-MB-231 and MTV/TM-011 cells (Figure 2B,C). As shown in Supplementary Figure S1, cell viability of MCF-10A was not affected by sauchinone treatment at 25 and 50 μM for 72 h, which suppressed the viability of breast cancer cells. These results were also confirmed in another normal bronchial epithelial cell line, Beas-2B. Based on these results, further experiments were performed by selecting a sauchinone concentration of 25 μM.

### Sauchinone inhibits migration and invasion of breast cancer cells

**Figure 3:**
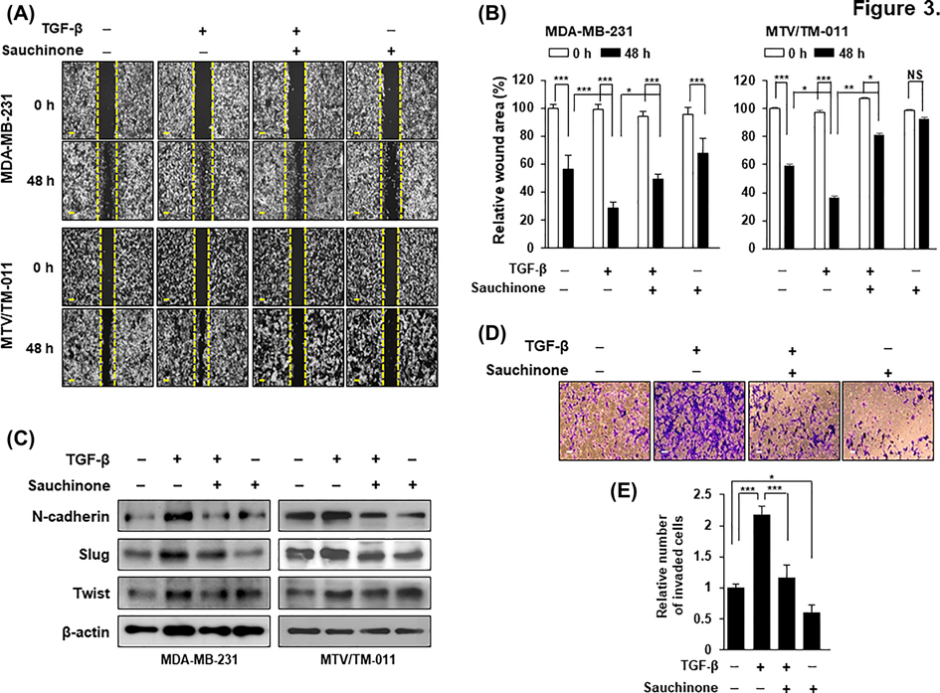
Effect of sauchinone on migration and invasion of breast cancer cells (**A**,**B**) MDA-MB-231 and MTV/TM-011 cells were seeded in 12-well plates and wounded by 1-ml pipette tip. The cells were incubated with TGF-β (10 ng/ml) or sauchinone (25 μM) for 48 h, separately or in combination. Scale bar = 200 µm. (**C**) Cells were pretreated with TGF-β (10 ng/ml) for 24 h and then incubated with sauchinone (25 μM) for another 24 h. Proteins were performed by Western blot analysis. (**D**,**E**) MTV/TM-011 cells were seeded in Matrigel-coated inserts and incubated with TGF-β (10 ng/ml) or sauchinone (25 μM) for 30 h, separately or in combination, followed by invasion assay. The invaded cells were counted and quantified using ImageJ. Scale bar = 200 µm. Data are presented using triplicate wells per group and statistical significance was determined by one-way ANOVA. *, *P* < 0.05; **, *P* < 0.01; ***, *P* < 0.001; NS, not significant.

To further elucidate the effect of sauchinone on cell mobility, we performed wound healing assay in MDA-MB-231 and MTV/TM-011 cells. As shown in Figure 3A,B, TGF-β-stimulated cells showed increased mobility after 48 h compared with control cells, but the increased cell migration was significantly inhibited by sauchinone treatment. Next, we investigated whether sauchinone inhibits TGF-β-induced EMT markers. TGF-β treatment of MDA-MB-231 and MTV/TM-011 cells increased the protein levels of N-cadherin, Slug, and Twist, which are involved in the mesenchymal cell phenotype. However, their levels were decreased by additional sauchinone treatment (Figure 3C). In addition, MTV/TM-011 cells stimulated with TGF-β showed a 2.27-fold increase in invasiveness compared with control cells, but the invasiveness of TGF-β-stimulated cells was remarkably suppressed by sauchinone treatment (Figure 3D,E). These results suggest that sauchinone inhibits the migration and invasion of breast cancer cells.

### Sauchinone inhibits MMP13 expression

**Figure 4:**
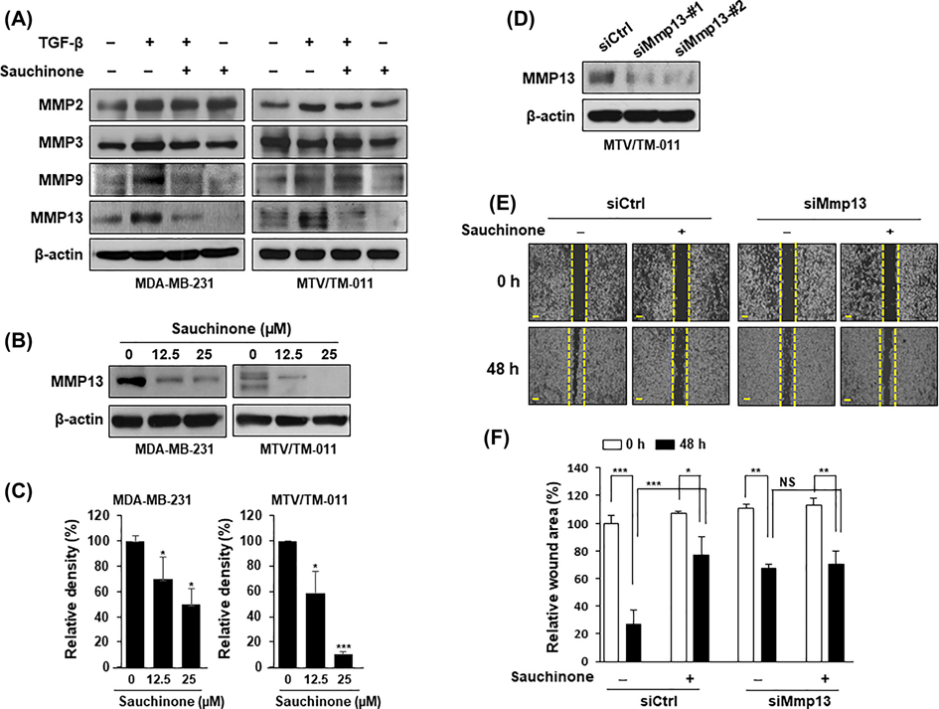
Effect of sauchinone on the expression of MMP13 (**A**) MDA-MB-231 and MTV/TM-011 cells were pretreated with TGF-β (10 ng/ml) for 24 h and then incubated with sauchinone (25 μM) for another 24 h. The effect of sauchinone on the expressions of MMP2, MMP3, MMP9, and MMP13 were assessed by Western blot analysis. (**B**,**C**) Cells were treated with sauchinone at different concentrations (12.5 and 25 μM) for 48 h and the lysed proteins were subjected to immunoblotting analysis. Protein expression was quantified using ImageJ. (**D**) The expression of MMP13 in MTV/TM-011 cells expressing siCtrl, siMmp13-#1, or siMmp13-#2 was evaluated by Western blot analysis. (**E**,**F**) MTV/TM-011 cells expressing siCtrl or siMmp13-#1 were seeded in culture-inserts and incubated with sauchinone (25 μM) for 48 h. Scale bar = 200 µm. Data are presented using triplicate wells per group and statistical significance was determined by one-way ANOVA. *, *P* < 0.05; **, *P* < 0.01; ***, *P* < 0.001; NS, not significant.

To explore the mechanisms underlying the regulation of migration and invasion in breast cancer cells following sauchinone treatment, we first screened representative MMPs (MMP2, MMP3, MMP9, and MMP13) via immunoblotting analysis. Interestingly, TGF-β stimulation up-regulated the expression of MMP13, which was markedly suppressed by sauchinone treatment in MDA-MB-231 and MTV/TM-011 cells (Figure 4A). Also, the expression of MMP13 was inhibited by sauchinone in a concentration-dependent manner (Figure 4B,C).

To evaluate MMP13 as a potent treatment target, we analyzed the effect of sauchinone on cell migration after knockdown of *Mmp13* in MTV/TM-011 cells. First, the knockdown of *Mmp13* expression by transferring siRNAs #1 and #2 was assessed via immunoblotting analysis (Figure 4D). Cell migration was attenuated by siMmp13-#1, but treatment of *Mmp13*-knockdown cells with sauchinone did not significantly inhibit cell migration compared with control cells (Figure 4E,F). This phenomenon was also observed using a cell invasion assay (Supplementary Figure S2). In addition, we performed an MMP13 rescue experiment with the *Mmp13*-knockdown breast cancer cells to ensure that MMP13 is a potential target for the inhibitory effect of sauchinone on breast cancer cell migration. The wound area was 78% in *Mmp13*-knockdown cells treated with sauchinone compared with that of the control cells. When MMP13 was overexpressed in *Mmp13*-knockdown cells, the wound area was increased to 89% by the sauchinone treatment (Supplementary Figure S3).

### Sauchinone suppresses MMP13 expression by regulating Akt-CREB activation

**Figure 5:**
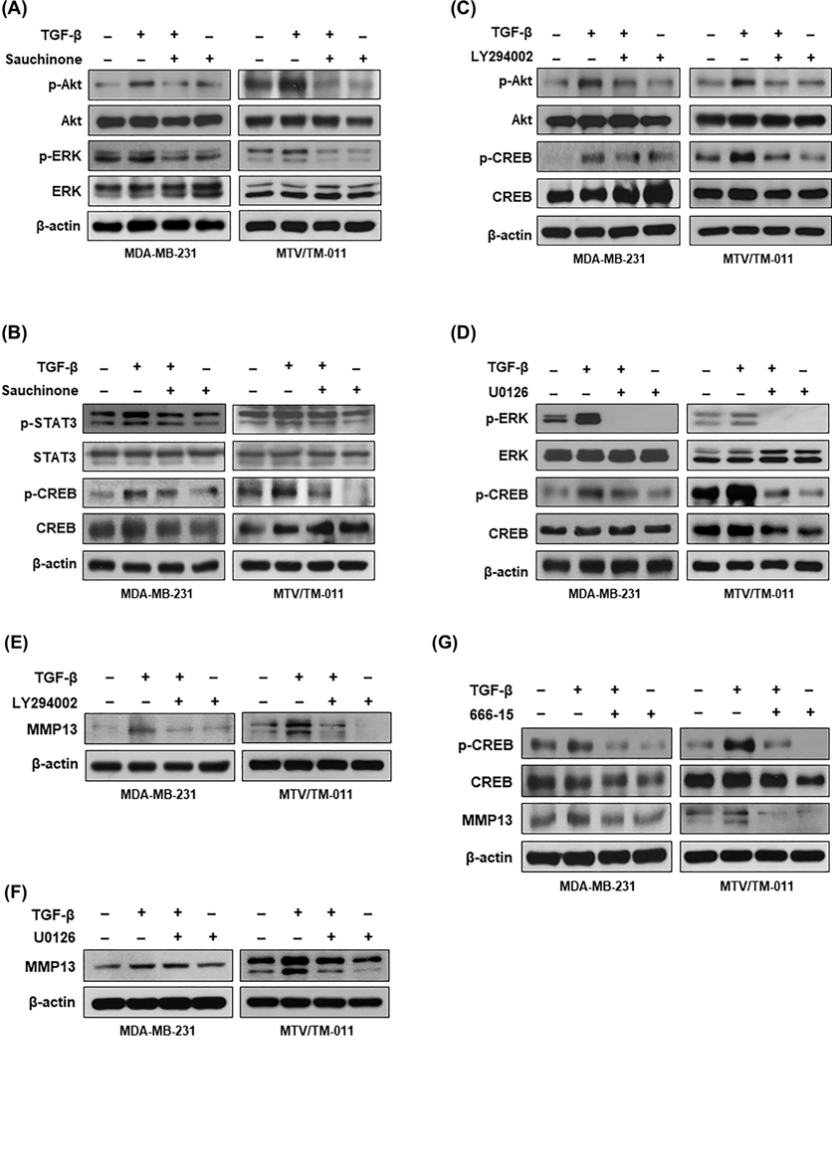
Effect of sauchinone on Akt-CREB signaling (**A**,**B**) MDA-MB-231 and MTV/TM-011 cells were cotreated with TGF-β (10 ng/ml) and sauchinone (25 µM) for 30 min. The lysates were immunoblotted with the indicated antibodies. (**C**,**D**) MDA-MB-231 and MTV/TM-011 cells were pretreated with LY294002 (25 µM) or U0126 (25 µM) for 2 h and then incubated with TGF-β (10 ng/ml) for another 30 min. The lysates were immunoblotted with the indicated antibodies. (**E**,**F**) Cells were pretreated with TGF-β (10 ng/ml) for 24 h and then incubated with LY294002 (25 μM) or U0126 (25 µM) for another 24 h. (**G**) Cells were cotreated with TGF-β (10 ng/ml) and 666-15 (5 µM) for 30 min (pCREB and CREB) or 48 h (MMP13).

Next, we investigated the intracellular signaling pathways mediating the inhibitory effect of sauchinone against MMP13 expression. As shown in Figure 5A, sauchinone treatment inhibited the increased phosphorylation of Akt and ERK by TGF-β in MDA-MB-231 and MTV/TM-011 cells. To further elucidate the underlying molecular mechanisms of sauchinone, we investigated the effect of sauchinone on the activation of STAT3 and CREB, which are the representative transcription factors involved in the regulation of MMP13 expression. Sauchinone treatment did not significantly affect the inhibition of STAT3 phosphorylation, but inhibited the phosphorylation of CREB (Figure 5B). Additionally, the phosphorylation of Akt and ERK increased by TGF-β mediated the activation of CREB. As shown in Figure 5C,D, the increased levels of CREB phosphorylation by TGF-β were reduced by treatment with an Akt kinase inhibitor (LY294002) or ERK kinase inhibitor (U0126).

To examine that the activation of Akt, ERK, or CREB regulates the expression of MMP13, MDA-MB-231 and MTV/TM-011 cells were treated with their respective inhibitors in the absence or presence of TGF-β. TGF-β treatment increased the expression of MMP13, which was markedly suppressed by LY294002 treatment (Figure 5E), but not significantly by U0126 treatment, particularly in MDA-MB-231 cells (Figure 5F). Similar to LY294002, the CREB inhibitor 666-15 also significantly inhibited the increase in MMP13 expression by TGF-β (Figure 5G). Taken together, these results suggest that sauchinone inhibits MMP13 expression by reducing the activation of Akt-CREB signaling.

## Discussion

*S. chinenesis* is known as one of the drugs for the treatment of edema and inflammatory diseases in Korea and China. To date, many studies have reported the pharmacological effects of *S. chinensis* extracts [30,31]. In particular, sauchinone is known to exhibit anti-inflammatory and anti-oxidant effects in various inflammatory models *in vitro* and *in vivo* [3,4,10,11]. However, to date, there are few studies on the anti-cancer effects of sauchinone. In particular, the effect of sauchinone on breast cancer cells and its underlying molecular mechanisms are not well known. In this study, sauchinone treatment inhibited the growth, migration, and invasion of breast cancer cells. To the best of our knowledge, this is the first report showing that sachinone inhibits MMP13 expression by suppressing Akt-CREB signaling in breast cancer cells.

Breast cancer is still one of the leading cancers worldwide, and even after treatment, there is a high risk of recurrence and metastasis [32]. When breast cancer spreads, it most commonly involves the bones, liver, lungs, and brain [33]. Overexpression of MMP13 in tumors is associated with aggressive tumor phenotype in breast cancer patients [34]. MMP13 expression is associated with increased breast cancer cell growth, EMT, migration and invasion [28,29,35]. In particular, MMP13 has been reported to promote the metastasis of breast cancer cells to organs such as bones and lungs [29,36,37]. Therefore, MMP13 is an important molecular target in breast cancer progression, highlighting the need to identify and develop clinically effective therapeutic agents.

Interestingly, several natural products have been reported to inhibit the expression of MMP13 [38–40]. The extract of *Urtica dioica*, a perennial herb, inhibited breast cancer cell migration by regulating microRNA-21 and several MMPs including MMP13 [39]. The ethanol extract of baked *Gardeniae fructus* inhibited the expression of MMP9 and MMP13, thereby suppressing the migration and invasion of human fibrosarcoma cells [40]. In this study, we also identified MMP13 as a new molecular target for sauchinone’s efficacy in inhibiting breast cancer cell growth, migration, and invasion.

Since cancer progression entails the expression of numerous genes, it is important to regulate the specific transcription factors directly involved in the expression of these oncogenes. Sauchinone has been reported to modulate the activity of various transcription factors underlying the anti-inflammatory, anti-oxidant, and anti-cancer effects. Sauchinone is known to inhibit the expression of cytokines and inflammatory molecules by suppressing the activity of transcription factors such as NF-κB [7,41] and STAT3 [42]. Additionally, sauchinone induces anti-inflammatory effects by increasing the activity of Nrf2, a representative transcription factor that regulates the expression of anti-oxidant enzymes [11,43]. Sauchinone has been reported to inhibit the growth of cancer cells by inhibiting the activity of transcription factors such as HIF-1α [16] and STAT3 [17]. CREB is a transcription factor involved in a variety of cellular processes, including cell proliferation, differentiation, immune response, and memory [44]. Sauchinone has not been shown to inhibit LPS-induced CREB activity in RAW264.7 cells [45]. However, our study demonstrated that sauchinone inhibits MMP13 expression by inhibiting the phosphorylation of CREB in breast cancer cells.

The breast cancer cell lines MDA-MB-231 and MTV/TM-011 were used in the present study. The MDA-MB-231 cell line is one of the most well-known triple-negative human breast cancer cells with strong metastatic properties [46]. The presence of the ERs in the MTV/TM-011 cell line has not yet been reported. MTV/TM-011 is a murine mammary carcinoma cell line that strongly induces lung metastasis [29]. As a result of this study, it is not possible to evaluate the effect of sauchinone on breast cancer cells depending on the state of ER. However, our findings clearly show that sauchinone treatment inhibits the growth, migration, and invasion of highly metastatic breast cancer cells.

In conclusion, our study is the first to establish that sauchinone suppresses the proliferation and invasion of metastatic breast cancer cells by inhibiting MMP13 expression. Here, we have identified the key molecules and molecular mechanisms underlying the effects of sauchinone in breast cancer cells. Sauchinone treatment inhibited MMP13 expression by down-regulating Akt-CREB signaling pathway in breast cancer cells. These results suggest that sauchinone may be a useful therapeutic agent for breast cancer treatment, and that the Akt-CREB-MMP13 axis may be a promising target for sauchinone.

## Supplementary Material

Supplementary Figures S1-S3

## Data Availability

All data are available from the corresponding author on reasonable request.

## Abbreviations

CREB: cAMP response element-binding protein
EMT: epithelial–mesenchymal transition
ER: estrogen receptor
HO-1: heme oxygenase-1
LPS: lipopolysaccharide
MMP: matrix metalloproteinase
Nrf2: nuclear factor erythroid 2–related factor 2
TGF-β: transforming growth factor-β
