# Supplementary material for: Sauchinone inhibits the proliferation, migration and invasion of breast cancer cells by suppressing Akt-CREB-MMP13 signaling pathway
Source: Biosci Rep. 2021 Oct 28;41(10):BSR20211067. doi: 10.1042/BSR20211067 (PMC8561391; doi:10.1042/BSR20211067)
Supplement: Supplementary Figures S1-S3 [file BSR-2021-1067_supp.pdf]

Supplementary Figure Legends

Supplementary Figure 1.

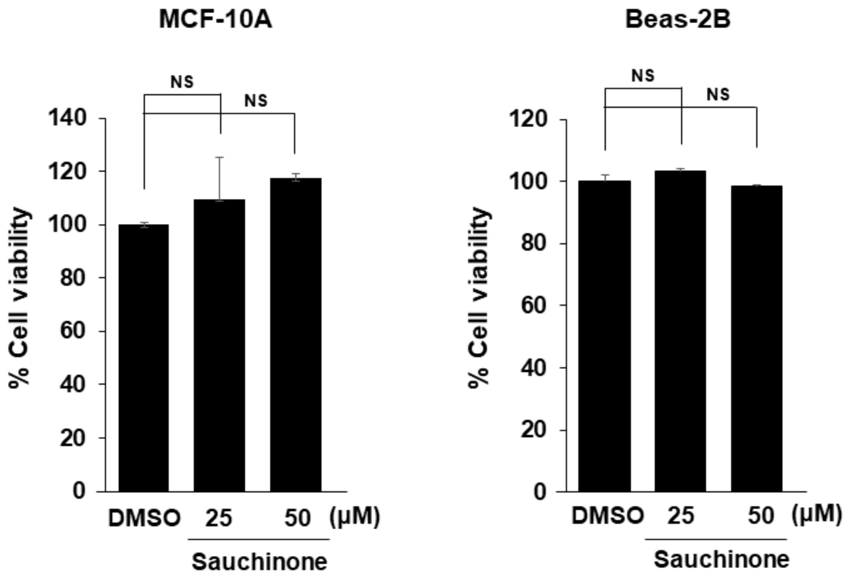

Fig. S1. Effect of sauchinone on cell viability of normal breast epithelial cells and normal bronchial epithelial cells. Cells were treated with sauchinone for 72 h, followed by MTS assay. Data are presented using triplicate wells per group and statistical significance was determined by one-way ANOVA. NS, not significant.

Supplementary Figure 2.

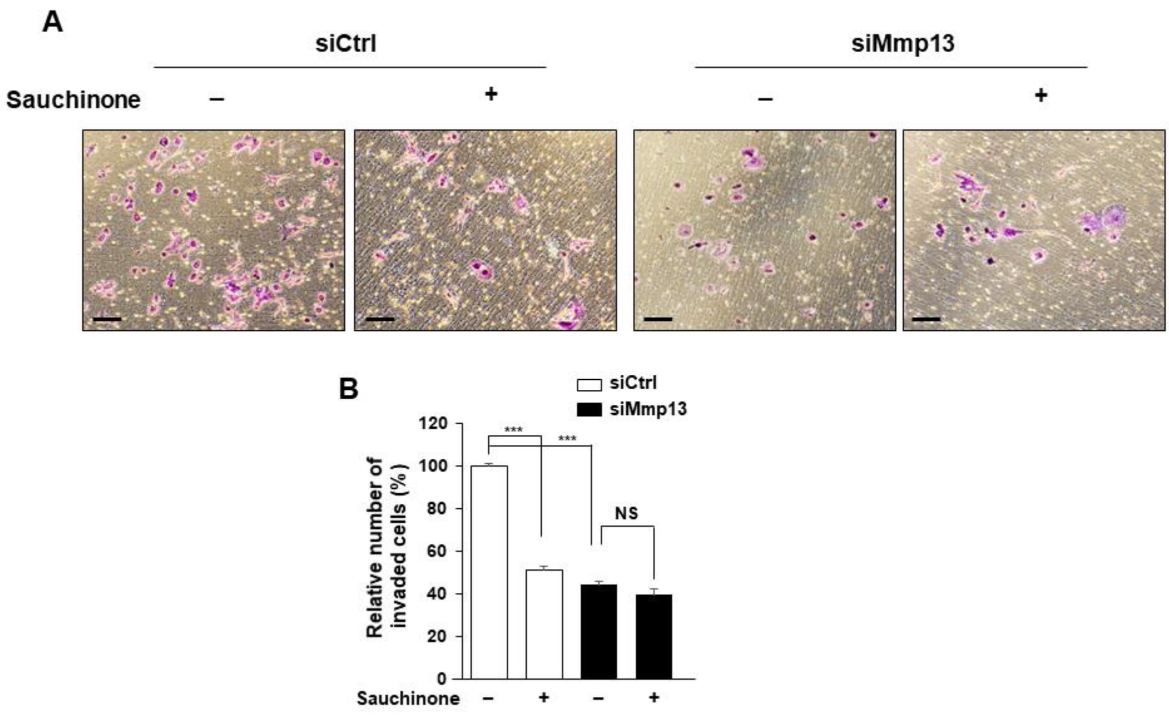

Fig. S2. Effect of sauchinone on cell invasion in *Mmp13*-knockdown breast cancer cells. **A,** MTV/TM-011 cells expressing siCtrl or siMmp13 were seeded in matrigel-coated inserts and incubated with sauchinone (25 μM) for 30 h, followed by invasion assay. **B,** The invaded cells were counted and quantified using Image J. Scale bar = 200 μm. Data are presented using triplicate wells per group and statistical significance was determined by one-way ANOVA. ***, $p < 0.001$; NS, not significant.

Supplementary Figure 3.

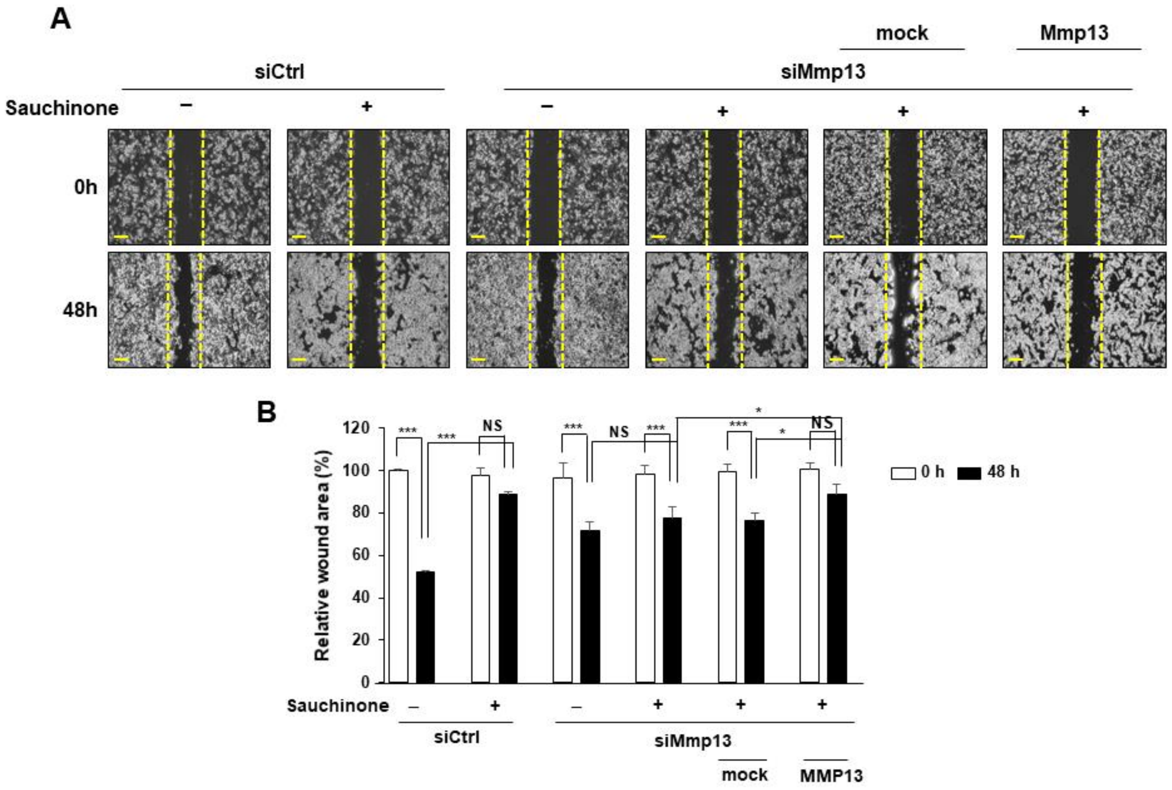

Fig. S3. Effect of sauchinone on cell migration following MMP13 overexpression in *Mmp13*-knockdown breast cancer cells. A, MTV/TM-011 cells were transfected with siCtrl or siMmp13 for 24 h. The *Mmp13*-knockdown cells were transfected with plasmids expressing mock or MMP13 for another 24 h. Cells were seeded in culture-inserts and incubated with sauchinone (25 μM) for 48 h. Scale bar = 200 μm. **B,** Data are presented using triplicate wells per group and statistical significance was determined by one-way ANOVA. *, p <0.05; ***, p < 0.001; NS, not significant.
